# Insights into shape of uveal melanoma: a comprehensive evaluation of clinical features, pathological features, and prognosis analysis

**DOI:** 10.3389/fmed.2025.1687291

**Published:** 2025-11-10

**Authors:** Heng Lou, Han Yue, Jiang Qian, Yingwen Bi, Xintong Lin, Haifeng Chen, Binbin Xu, Ruiqi Ma, Kang Xue, Jie Guo

**Affiliations:** 1 Eye Institute and Department of Ophthalmology, Eye and ENT Hospital, Fudan University, Shanghai, China; 2 Key Laboratory of Myopia and Related Eye Diseases, NHC, Shanghai, China; 3 Key Laboratory of Myopia and Related Eye Diseases, Chinese Academy of Medical Sciences, Shanghai, China; 4 Department of Pathology, Eye and ENT Hospital, Fudan University, Shanghai, China

**Keywords:** uveal melanoma, echography, tumor shape, prognosis, metastasis

## Abstract

**Background:**

Many patients with uveal melanoma (UM) cannot receive laboratory analysis due to eye-preserving treatment, biopsy risks or costs. The study is to evaluate correlation between tumor shape and a series of metastasis risks in UM, and to assess the predictive value of tumor shape classification.

**Methods:**

Four hundred thirty-nine UM patients undergoing enucleation were included in the study. Standardized echography was utilized to document selected tumor characteristics. Tumors were categorized into five distinct shape groups: mushroom, dome, lobulated, diffuse, and irregular. Clinical data, tumor thickness, largest basal diameter (LBD), American Joint Committee on Cancer (AJCC) stage, pathological results, and survival status were collected and comparatively analyzed across the different tumor shapes. Survival analysis was carried out with both Cox hazard regression and Kaplan–Meier log rank test.

**Results:**

The 439 UM cases were classified as mushroom in 164 (37.4%), dome in 129 (29.4%), lobulated in 62 (14.1%), diffuse in 11 (2.5%) and irregular in 73 (16.6%). Significant differences were observed in tumor thickness, LBD, cell type, ciliary body involvement (CBI), extraocular extension, and AJCC stage across these shape categories. Regardless of tumor size and AJCC stage, mushroom-shaped melanoma exhibited the most favorable prognosis, irregular-shaped melanoma demonstrated the worst prognosis.

**Conclusion:**

Tumor shape could be defined noninvasively and dependably using echography. Shape classification in UM provides an independent variable to improve the clinical prognostication.

## Introduction

Uveal melanoma (UM) stands as the most common primary ocular malignancy among adults. Despite continuous improvements in diagnosis and management, UM still has a high tendency to metastasize resulting in high mortality (1–5).

Improved prognostication for UM enables the identification of patients at high risk for metastasis, facilitating targeted screening and potential adjunctive/adjuvant systemic therapy. Various clinical, pathologic and cytogenetic features of UM are associated with metastatic risk. Clinical factors mainly include age, tumor size, ciliary body involvement (CBI) and American Joint Commission on Cancer (AJCC) staging system (6–8). Histopathologic factors mainly include cell type, extraocular extension and mitotic activity (9–11). More recently, cytogenetic factors have gained popularity (11–14). Chromosome 3 loss, 8q gain are associated with poor prognosis (15). Based on gene expression profiling (GEP), Class I UM are unlikely to metastasize, whereas Class II predict a higher rate of metastasis (16, 17). However, pathologic and cytogenetic factors are sometimes restricted in clinical practice: for eye-preserving therapy has become more frequently, or concerns about biopsy risks or costs, a considerable number of patients may lack laboratory analysis about metastatic risks. It is still important to improve clinical prognostic system.

Tumor size, including thickness and largest basal diameter (LBD), is key clinical factor in assessing prognosis of UM (18). Tumor shape also showed a certain effect on metastatic risk. Ultrasonography categorizes UM into several distinct shapes (19), diffuse melanoma poses a significant risk for metastasis (8, 20) dome-shaped melanoma showed some association with a more favorable prognosis (21, 22). However, systemic study about tumor shape and metastatic risks is rare.

In this study, we investigated the association between tumor shape and other metastasis risk factors. Additionally, we examined the predictive significance of tumor shape.

## Methods

This retrospective study was conducted at Eye & ENT Hospital of Fudan University. The study was approved by the Ethics Committee of the hospital and he process of data collection and analysis adhered to the Declaration of Helsinki.

The inclusion criteria included patients who were diagnosed with UM and underwent enucleation between April 2003 and March 2023. Only patients without prior treatment or confirmed metastasis were included. Iris melanomas were excluded.

The clinical data including age and gender were collected, along with the survival status (recorded as either melanoma-free survival or metastasis present), if applicable. Tumor staging adhered to the AJCC Classification (8th edition) (23).

The echographic records included tumor location, thickness, LBD, shape, extrascleral extension (EXE), CBI and relationship to optic disc (19). All ultrasound images were retrospectively and independently reviewed by two ultrasound physicians with over 8 years of experience in ocular oncology. Each physician classified the tumor shape according to the five-category scheme while blinded to the patient’s clinical outcome. Initial inter-observer agreement was assessed. For cases with discrepant classifications (which constituted approximately 8% of the total), a consensus meeting was held where the images were re-examined jointly, discussed with reference to standard diagnostic criteria, and a final consensus classification was reached. Tumor was categorized into five shape groups, primarily adhering to the Collaborative Ocular Melanoma Study (COMS) definitions (8, 19, 24, 25): (1) mushroom; (2) dome; (3) diffuse, flat tumor with thickness ≤20% of LBD (4) lobulated (5) irregular, those not fitting in the above shapes, usually with irregular contour. The presence of CBI was judged in combination with ultrasound biomicroscope (UBM) if applicable.

The histopathological assessment was conducted by a single experienced pathologist to determine the cell type, presence of CBI, optic nerve invasion and EXE. EXE was defined as any tumor extension beyond the outer surface of the sclera. Optic nerve invasion was described as the infiltration of tumor cells into the optic nerve including prelaminar and postlaminar regions.

### Statistical analysis

Statistical analyses were performed using SPSS 22.0 and R version 4.4.2. Continuous data were presented as mean and standard deviation (SD) (with a 95% confidence interval, CI). Categorical variables were presented as numbers and percentages. Kruskal–Wallis *H* test with Bonferroni’s *post hoc* analysis was employed for non-normally distributed data or those lacking homogeneous variance across multiple groups. One-way ANOVA was used to compare multiple groups with normal distribution and homogeneity variance. Pearson’s chi-square and Fisher’s exact tests were used for categorical data analysis. Pairwise comparisons among subgroups were made and the adjusted *p* value was considered statistically significant according to Bonferroni corrections. Overall survival was analyzed using the Kaplan–Meier (KM) estimator of survival curves, with group differences evaluated via the log-rank test. Receiver operating characteristic (ROC) curve analysis was used to determine the threshold LBD. Univariate Cox analysis was used to investigate the relationship between the patient’s age, gender and tumor characteristics with risk of metastasis. Multivariable Cox analysis was employed to examine the association of significant covariates with metastasis. *p* < 0.05 was considered statistically significant.

## Results

### Baseline characteristics of patients and tumors

**Figure 1:**
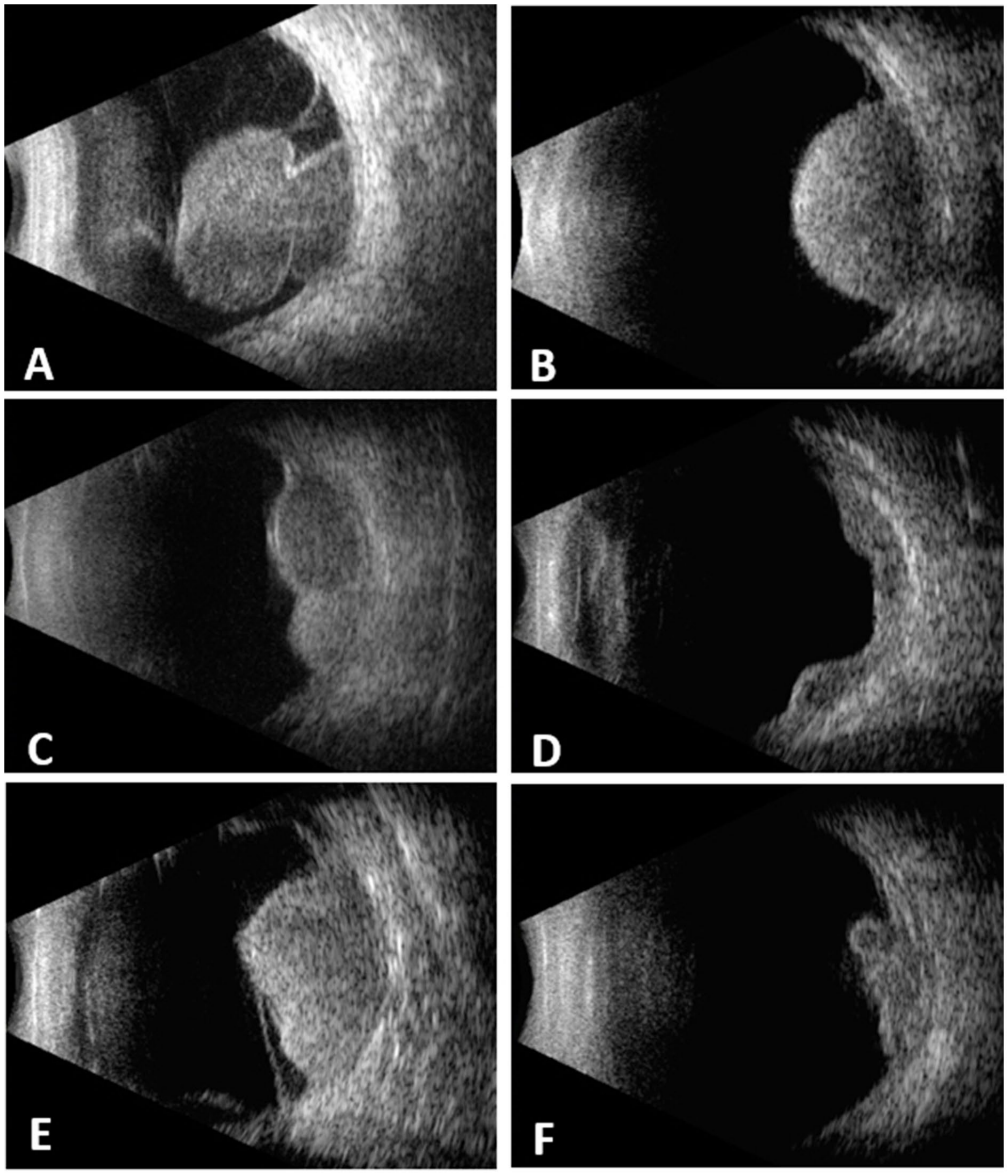
Choroidal melanomas with different configurations demonstrated on B-scan echograms. **(A)** mushroom. **(B)** dome. **(C)** lobulated. **(D)** diffuse. **(E)** irregular. **(F)** irregular.

A total of 439 cases were included. 218 (49.7%) were female and 221 (50.3%) were male. The mean age at diagnosis was 51.4 ± 13.7 years (7–83). The tumor shape was mushroom in 164 (37.4%), dome in 129 (29.4%), lobulated in 62 (14.1%), diffuse in 11 (2.5%) and irregular in 73 (16.6%) (Figure 1). The mean tumor thickness was 8.9 ± 3.1 mm (2.7–21.0) and mean LBD was 13 ± 4 mm (4.9–25.0).

### Association between tumor shape and metastatic risk factors

The association of tumor shape with metastatic risk factors is listed in Table 1. Tumor thickness was highest in irregular and mushroom groups, and was lowest in diffuse group. The lobulated and diffuse groups exhibited the widest LBD.

**Table 1:** The association between tumor shape and various metastatic risk factors.

Characteristic	Tumor shape					
	Mushroom (*n* = 164)	Dome (*n* = 129)	Lobulated (*n* = 62)	Diffuse (*n* = 11)	Irregular (*n* = 73)	*P* value
Age at diagnosis	51 ± 13 (22–82)	49 ± 13 (15–77)	54 ± 13 (7–80)	53 ± 14 (25–71)	54 ± 15 (14–83)	0.06^#^
Gender						0.007^*^
Female	69 (42.1)^a^	79 (61.2)^b^	34 (54.8)^ab^	3 (27.3)^ab^	33 (45.2)^ab^	
Male	95 (57.9)^a^	50 (38.8)^b^	28 (45.2)^ab^	8 (72.7)^ab^	40 (54.8)^ab^	
Thickness	9.8 ± 2.6 (3.5-20)^a^	7.2 ± 2.7 (2.7–17.1)^bc^	8.2 ± 2.4 (3.1–15.5)^b^	4.3 ± 1.5 (2.7–7.7)^c^	11.1 ± 3.3 (4.8-21)^a^	<0.001^##^
LBD	10.9 ± 2.5 (5.6–19.6)^a^	12.4 ± 3.1 (6.4–24.2)^b^	17.1 ± 3.5 (11.2-25)^c^	16.7 ± 4.0 (12-24)^cd^	14.4 ± 4.6 (4.9-25)^d^	<0.001^##^
Thickness/LBD	0.9 ± 0.2 (0.4–1.7)^a^	0.6 ± 0.2 (0.3–1.0)^b^	0.5 ± 0.1 (0.3–1.0)^bc^	0.3 ± 0.1 (0.1–0.4)^c^	0.8 ± 0.2 (0.3–1.5)^a^	<0.001^##^
Cell type						<0.001^**^
Spindle	87 (53)^a^	60 (46.5)^a^	15 (24.2)^b^	0 (0)^b^	15 (20.6)^b^	
Mixed	64 (39)^a^	51 (39.5)^a^	35 (56.5)^a^	7 (63.6)^a^	39 (53.4)^a^	
Epithelioid	13 (8)^a^	18 (14)^ab^	12 (19.3)^ab^	4 (36.4)^b^	19 (26)^b^	
CBI						<0.001^*^
No	154 (93.9)^a^	107 (82.9)^b^	40 (64.5)^c^	6 (54.5)^bc^	33 (45.2)^c^	
Yes	10 (6.1)^a^	22 (17.1)^b^	22 (35.5)^c^	5 (45.5)^bc^	40 (54.8)^c^	
EXE						<0.001^**^
No	163 (99.4)^a^	126 (97.7)^ab^	59 (95.2)^ab^	7 (63.6)^c^	66 (90.4)^bc^	
Yes	1 (0.6)^a^	3 (2.3)^ab^	3 (4.8)^ab^	4 (36.4)^c^	7 (9.6)^bc^	
Optic nerve invasion						<0.001^*^
No	151 (92.1)^a^	110 (85.3)^ab^	47 (75.8)^b^	7 (63.6)^b^	62 (84.9)^ab^	
Yes	13 (7.9)^a^	19 (14.7)^ab^	15 (24.2)^b^	4 (36.4)^b^	11 (15.1)^ab^	
AJCC tumor size						<0.001^**^
*T*1	6 (3.7)^a^	13 (10.1)^a^	0 (0)^a^	0 (0)^a^	3 (4.1)^a^	
*T*2	56 (34.1)^ab^	58 (45.0)^b^	7 (11.3)^c^	6 (54.5)^b^	12 (16.4)^ac^	
*T*3	90 (54.9)^a^	51 (39.5)^ab^	35 (56.4)^a^	0 (0)^b^	34 (46.6)^a^	
*T*4	12 (7.3)^a^	7 (5.4)^a^	20 (32.3)^b^	5 (45.5)^b^	24 (32.9)^b^	
AJCC stage						<0.001^**^
I	6 (3.7)^a^	12 (9.3)^a^	0 (0)^a^	0 (0)^a^	3 (4.1)^a^	
II	139 (84.7)^a^	94 (72.9)^a^	31 (50.0)^b^	2 (18.2)^b^	22 (30.1)^b^	
III	19 (11.6)^a^	23 (17.8)^a^	31 (50.0)^b^	9 (81.8)^b^	48 (65.8)^b^	

LBD, largest basal tumor diameter; CBI, ciliary body involvement; EXE, extraocular extension; AJCC, American Joint Committee on Cancer.

a, b, c, d: The same letter means the difference is not statistically significant, and the different letter indicate a statistically significant difference between the two groups.

^*^Pearson chi-square test (significant *p* value < 0.05).

^**^Fischer’s exact test (significant *p* value < 0.05).

^#^One way ANOVA test (significant *p* value < 0.05).

^##^Kruskal–Wallis *H* test (significant *p* value < 0.05).

The proportion of spindle cell type tumor was highest in mushroom group; no spindle cell type tumor was found in diffuse group. Additionally, the thickness and LBD among different cell types was compared within each shape group, and no significant difference was found (*p* = 0.07–0.89).

CBI was noted in 99 cases, with the lowest incidence in mushroom group and the highest in irregular group. The diffuse and irregular group showed a significantly higher incidence of EXE compared to mushroom group. Optic nerve invasion was noted in 62 cases, with significantly higher incidences in lobulated and diffuse groups compared to mushroom group. According to AJCC criteria, tumors were classified as *T*1 (5.0%), *T*2 (31.7%), *T*3 (47.8%), and *T*4 (15.5%), corresponding to stage classifications of I (4.8%), II (65.6%), and III (29.6%). Mushroom-shaped and dome-shaped tumors predominantly fell into stage II, diffuse-shaped and irregular-shaped tumors were more likely to fall into stage III.

### Metastatic and survival rates in different shape classification

Follow-up data were available for 282 cases, of who 72 developed metastases. The mean duration from initial diagnosis to the onset of metastasis was 46.2 ± 36.8 months. For patients who did not develop metastasis, the average interval from initial diagnosis to the last follow-up was 77.1 ± 49.4 months.

The overall metastasis-free survival rates at 5 and 10 years were 81.9 and 74.5%, respectively. At the 5-year follow-up, metastasis-free survival rates were 93.2% in mushroom group, 89.5% in dome group, 81.8% in lobulated group, 71.4% in diffuse group, and 40.9% in irregular group. At the 10-year follow-up, metastasis-free survival rates were 92.2% in mushroom group, 78.9% in dome group, 75.8% in lobulated group, and 22.7% in irregular group. Of the 7 cases in diffuse group, 2 patients died from metastasis (at 21 and 34 months), metastasis developed in 1 patient at 77 months, four were alive without metastasis (followed from 28 to 50 months).

### Prognosis analysis on tumor shape classification

Table 2 presents the results of the univariate Cox Regression analysis. In the analysis, tumors exhibiting dome, lobulated, diffuse or irregular shapes (compared to the mushroom shape) showed significant associations with metastasis (*p* < 0.05). Gender, tumor thickness, LBD, cell type, CBI, EXE, optic nerve invasion and AJCC stage were significantly associated with metastasis (*p* < 0.05).

**Table 2:** Uni-variate Cox Regression, hazard for metastasis (*n* = 282).

Variable	Univariate		
	HR	95% CI	*P* Value
Age	1.015	0.998–1.032	0.091
Gender (female vs. male)	1.624	1.010–2.611	0.045
Thickness (mm)	1.122	1.048–1.201	0.001
LBD (mm)	1.175	1.119–1.234	<0.001
Shape			
Dome vs. mushroom	2.937	1.293–6.670	0.01
Lobulated vs. mushroom	4.826	1.803–12.916	0.002
Diffuse vs. mushroom	8.541	1.778–41.033	0.007
Irregular vs. mushroom	16.018	7.376–34.787	<0.001
Cell type (nonspindle vs. spindle)	2.006	1.242–3.239	0.004
CBI (yes vs. no)	4.762	2.952–7.683	<0.001
EXE (yes vs. no)	5.095	2.713–9.568	<0.001
Optic nerve invasion (yes vs. no)	2.023	1.184–3.456	0.01
AJCC stage			
II vs. I	2.583	0.352–18.943	0.351
III vs. I	10.844	1.486–79.125	0.019

LBD, largest basal tumor diameter; CBI, ciliary body involvement; EXE, extraocular extension.

**Figure 2:**
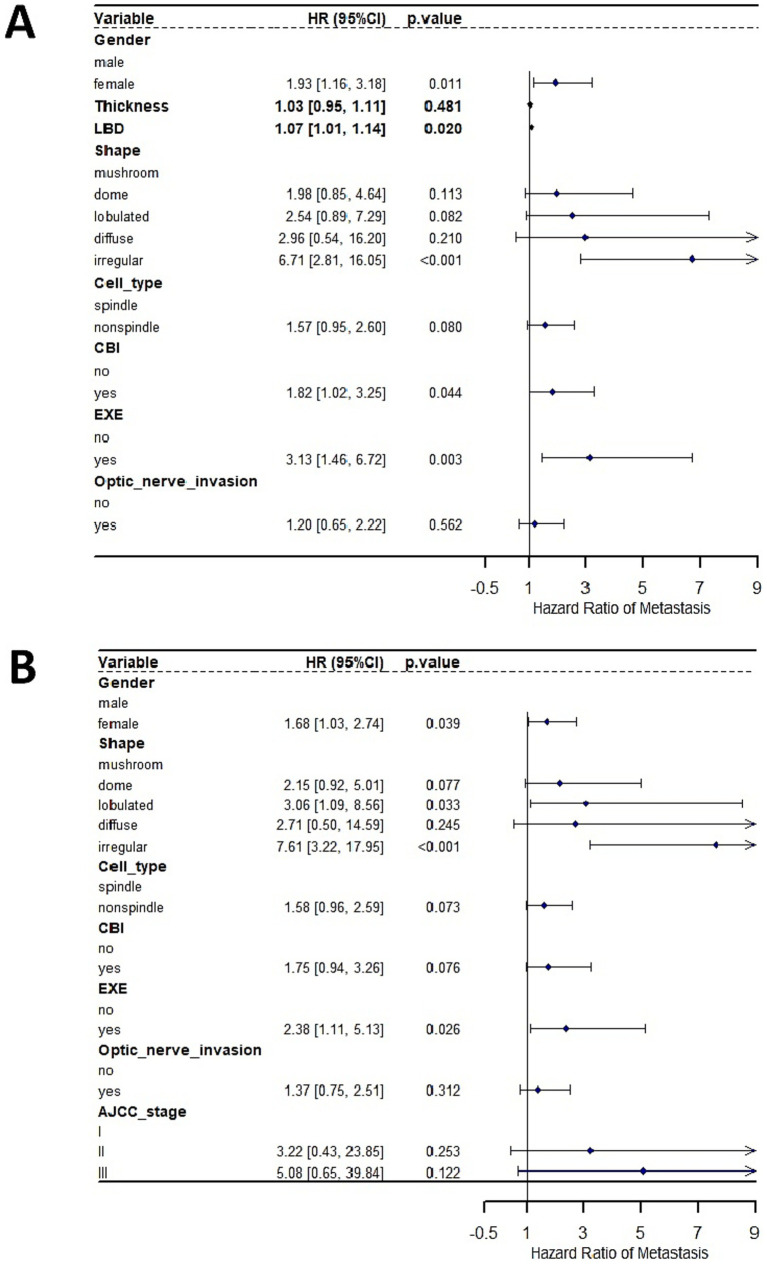
Multivariate Cox Regression, hazard for metastasis.

Given that tumor thickness, LBD and AJCC stage were all measures of tumor size, we analyzed their associations with metastasis using two independent multivariate Cox Regression analysis models. In a model where tumor thickness and LBD were used as measure of tumor size, multivariate Cox Regression analyses showed that irregular tumor shape (compared to mushroom shape), gender, increasing LBD, CBI, and EXE emerged as significant independent prognostic factors predicting the development of metastasis (Figure 2A). No significant interaction between LBD and tumor shape was observed (*p* = 0.078). In an alternative model where AJCC stage was used as a surrogate of tumor size, multivariate Cox Regression analyses showed that lobulated and irregular tumor shape (compared to mushroom shape), gender, and EXE emerged as significant independent prognostic factors predicting the development of metastasis (Figure 2B).

**Figure 3:**
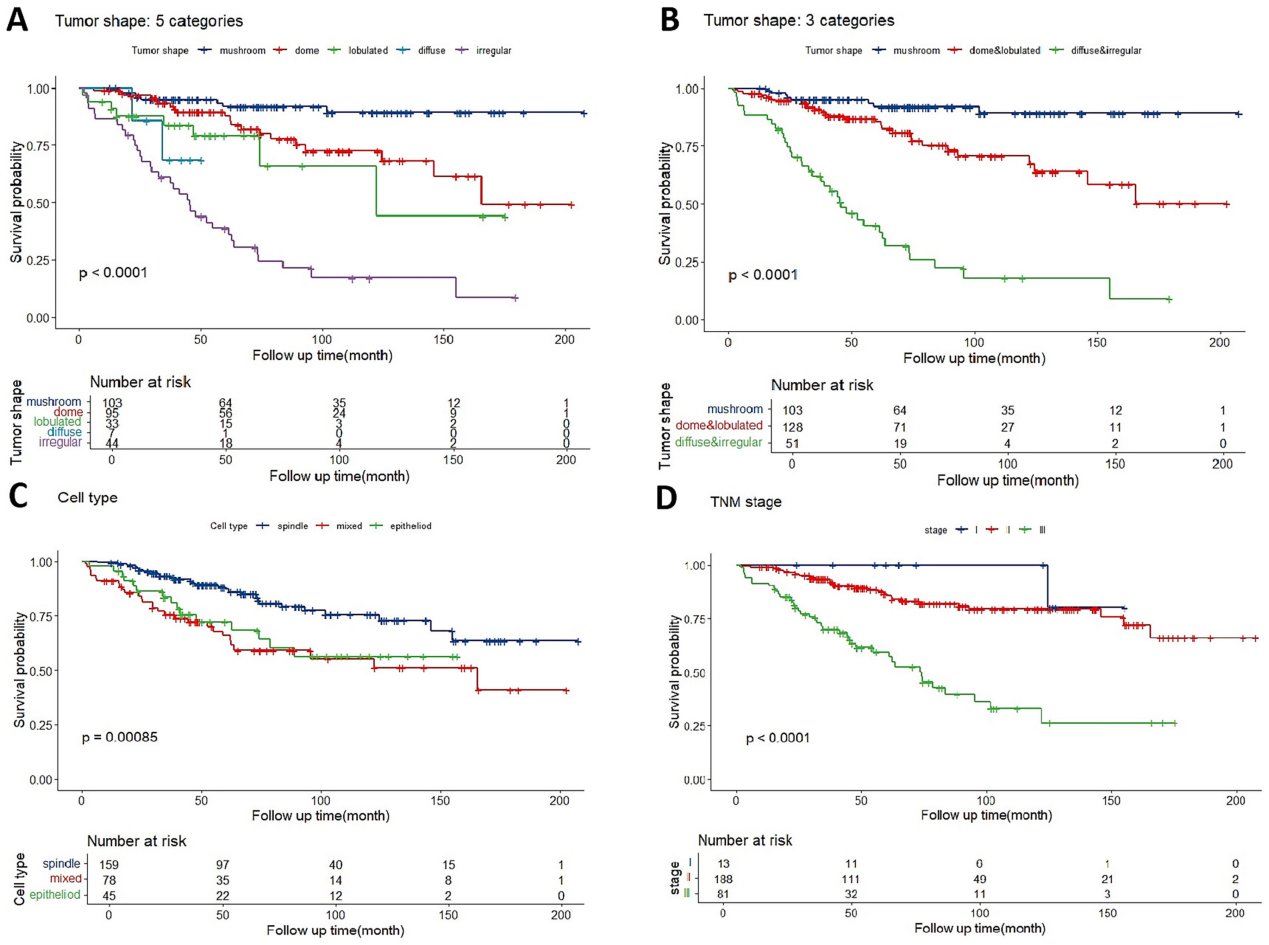
**(A)** Kaplan–Meier estimator of overall metastasis-free survival based on five classifications of tumor shape. **(B)** Metastasis-free survival curves are shown based on optimized categorization of tumor shape into three categories. **(C)** Overall metastasis-free survival based on three categories of AJCC stage. **(D)** Overall metastasis-free survival based on three categories of cell type.

Kaplan–Meier analysis was used to identify and integrate prognostically redundant categories in the five groups to optimize the tumor shape model (Figure 3A). Mushroom-shaped melanoma had the most favorable prognosis, whereas irregular-shaped melanoma had the worst. Furthermore, no significant differences were observed between the dome and lobulated groups (*p* = 0.203), lobulated and diffuse groups (*p* = 0.53), or diffuse and irregular groups (*p* = 0.379). Then the dome + lobulated and diffuse + irregular groups were merged, resulting in three distinct categories (Figure 3B). The optimized tumor shape categories demonstrated superior prognostic accuracy when compared to AJCC stage (Figure 3C) and cell type (Figure 3D) categories, at every follow-up point, the tumor shape model maintained a greater separation between metastatic risk groups than did the cell type and AJCC stage categories.

**Figure 4:**
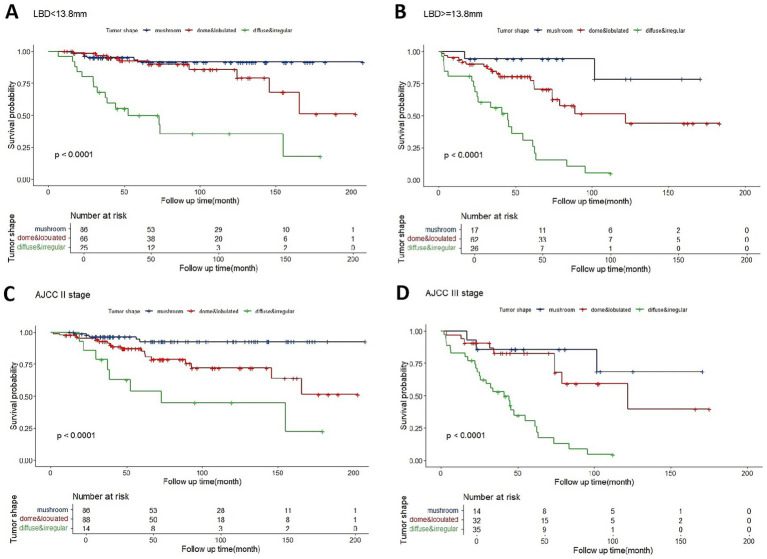
Kaplan–Meier estimator of overall metastasis-free survival for tumors with LBD < 13.8 mm **(A)**, LBD ≥ 13.8 mm **(B)**, AJCC stage II **(C)**, or AJCC stage III **(D)** based on optimized categorization of tumor shape into three categories.

The ROC curve indicated that a LBD threshold of ≥13.8 mm predicted 5-year metastasis. For UM with LBD less or not less than 13.8 mm, mushroom-shaped tumors consistently showed a significantly lower metastatic risk, while diffuse and irregular tumors exhibited a higher metastatic risk throughout the follow-up period (Figures 4A,B). Dome or lobulated tumors with LBD less than 13.8 mm initially had a similar metastatic risk to mushroom-shaped tumors, but their risk increased after 89 months (Figure 4A). Similar trends were observed in AJCC stage II and III tumors (Figures 4C,D). There were only 13 patients who fell into AJCC stage I (three mushroom-shape, eight dome-shape, and two irregular-shape) in the current study, which was insufficient for survival curve analysis.

### Consistency between echographic findings and pathologic outcomes

The comparison of tumor shape between echograms and histologic slides revealed an agreement rate of 70.6%. Table 3 summarizes the consistency between echographic findings and pathological outcomes.

**Table 3:** Association between echographic findings and pathological outcomes in uveal melanoma patients.

Echographic findings		Histopathogical outcomes	
Extrascleral extension	No.		No. (%)
Definite	4	Macroscopic	4 (100%)
		Microscopic	0 (0%)
		None	0 (0%)
Possible	9	Macroscopic	1 (11.1%)
		Microscopic	3 (33.3%)
		None	5 (55.6%)
None	426	Macroscopic	0 (0%)
		Microscopic	10 (2.3%)
		None	416 (97.7%)
Optic nerve invasion	No.		No. (%)
Cover optic disc	22	Postlaminar invasion	7 (31.8%)
		Prelaminar invasion	12 (54.5%)
		None	3 (13.7%)
Contact optic disc	51	Postlaminar invasion	1 (2%)
		Prelaminar invasion	33 (64.7%)
		None	17 (33.3%)
None	366	Postlaminar invasion	1 (0.3%)
		Prelaminar invasion	8 (2.2%)
		None	357 (97.5%)
Cilary body involvement	No.		No. (%)
Positive	89	Positive	80 (89.9%)
		Negative	9 (10.1%)
Negative	350	Positive	19 (5.4%)
		Negative	331 (94.6%)

## Discussion

Various factors have been employed to predict metastasis in UM, while histopathological and genomic factors represent a substantial stride towards the development of precise prognostic markers, they are associated with invasive operation, extra costs and concerns on intratumoral heterogeneity (26–29), further research is warranted to develop non-invasive factors to improve the current clinical prognosis assessment, such as AJCC staging system or Liverpool Uveal Melanoma Prognosticator Online (LUMPO) (30–32).

Measurement of tumor size is the cornerstone of UM prognostication. Ocular echography is the most valuable tool for determining tumor size and configuration. Numerous studies have shown a strong correlation between tumor LBD and reduced survival probability (33, 34). Tumor thickness was considered to be an survival predictor except in diffuse melanoma (2), but it does not appear to be a significant independent risk factor in multivariate analyses (33). Besides LBD, the cell type significantly influences prognosis in UM (11, 35). EXE and CBI has also been recognized as adverse prognostic factors (36–40). Although optic nerve invasion is relatively infrequent in UM, postlaminar invasion has consistently been linked to a poorer prognosis and increased melanoma-related mortality (41, 42). The AJCC staging system has been widely adopted to assess metastatic risk (6). More recently, tumor volume classification has emerged as one of the strongest prognostic indicators in UM (18).

However, tumor configuration had rarely been involved in the pathologic or prognostic studies of UMs. In the COMS study, the most common tumor shape was dome (60%), followed by mushroom (27%), then lobulated (6%). The epithelioid cell type tumors were more prone to exhibit a lobulated shape (43). These findings align closely with another study, which categorized UMs into four shapes: the tumor had hemispheric-shape in 61.1%, mushroom-shape in 27.9%, flat-shape in 2.6%, and irregular-shape in 8.4% (22). In our study, we categorized tumors into five shapes based on echograms. Our findings revealed a predominance of mushroom-shaped and dome-shaped tumors. This discrepancy with previous reports could potentially be attributed to differences in tumor size. Dome-shaped tumors are more commonly observed in small and medium-sized UMs. Importantly, our study exclusively included UM patients who underwent enucleation, and the tumor size (as indicated by tumor thickness or the proportion of medium and large-sized tumors) exceeded that of the other two studies (22, 43).

In our study, the direct concordance rate between the pre-operative ultrasound tumor shape and the post-operative pathological assessment was 70.6%. This discrepancy of approximately 30% warrants consideration regarding the potential influences of the pathological processing on morphological interpretation. The enucleation procedure itself, followed by formalin fixation and paraffin embedding, can introduce tissue shrinkage and architectural distortions. Furthermore, the pathological evaluation is inherently dependent on the plane of sectioning. A single histological section may not perfectly capture the three-dimensional, *in vivo* geometry of the tumor as visualized by ultrasound; for instance, an oblique cut through a dome-shaped tumor might yield a flat or irregular profile on a glass slide. Therefore, while perfect one-to-one correspondence with pathology is challenging, the pre-operative B-scan provides a reliable and clinically relevant representation of the tumor’s morphology.

In this study, distinct clinical and pathological features have been demonstrated in UMs of different shapes. Dome-shaped melanomas are characterized by relatively smaller tumor dimensions, a higher prevalence of spindle cell types, and a lower incidence of CBI and extraocular invasion. In contrast, lobulated, diffuse, and irregular melanomas exhibit larger tumor sizes, a higher proportion of non-spindle cell types, and an increased likelihood of CBI and extraocular invasion. Notably, diffuse melanomas display the highest proportion of epithelioid cell types and the greatest risk of EXE and optic nerve invasion, whereas irregular melanomas are most predisposed to CBI. Despite their significant thickness, mushroom-shaped melanomas are predominantly composed of spindle cell types and exhibit the lowest incidence of CBI and extraocular invasion. AJCC staging further indicated that diffuse and irregular melanomas are associated with more advanced stages compared to mushroom and dome-shaped melanomas. These prognostic factors exhibit notable variations among UMs with different shape classifications.

In our study, the multivariable analysis pinpointed tumor shape classification as an independent prognostic factor, it even surpassed other variables in predicting metastasis. Mushroom-shaped melanomas had the most favorable prognosis, while irregular-shaped ones had the worst, despite similar tumor sizes between the two groups. Kaplan–Meier analysis further validated the superior prognostic accuracy of tumor shape classification over cell type classification and AJCC stage. Furthermore, this trend was observed in tumors with both larger and smaller LBD, as well as in AJCC stage II and III tumors. Shields et al. (8) supposed that diffuse UM showed a higher probability of metastasis and death. Liu et al. (22) reported survival rates at 5 years varied significantly among different tumor shapes. Rusakevich et al. (21) found an significant association between tumor shape and PRAME, a melanoma marker associated with increased metastatic risk in UMs. In our study, dome-shaped tumors had a less optimistic prognosis, likely attributed to their larger size in our cohort.

Growth pattern might decide the formation of tumor morphology. Mushroom-shaped melanomas are characterized by the rupture of Bruch’s membrane (44). Actually, rupture of Bruch’s membrane is prevalent in most UMs, with a higher proportion in non-spindle or large-sized melanomas (45), which has been correlated with an increased risk of metastasis (46), but the mechanism remains unclear. Mushroom-shaped melanomas exhibit a “breaking” growth pattern, Bruch’s membrane undergoes early but localized rupture, likely due to a mechanical compression, resulting in the smallest LBD, potentially suggesting less aggressiveness. In contrast, irregular-shaped melanomas display a “multi-protuberance” growth pattern, with extensive destruction of Bruch’s membrane, potentially mediated by proteases (47), which may serve as a potential mechanism for metastasis. Regarding dome-shaped tumors, especially those of smaller sizes, the possibility that they may serve as precursors to tumors with different shapes cannot be overlooked.

Although it’s really challenging to confirm tiny extrascleral invasion by echography or whether the tumor has invaded optic nerve, our study indicated that echography could provide crucial clues to estimate EXE, CBI and optic nerve invasion in UM.

This study still had some limitations. Firstly, the cohort is limited to UM patients who underwent enucleation, and the sample size is relatively small. A larger cohort that includes patients with smaller tumors and/or undergoing eye-preserving therapies would enhance the accuracy of our findings. Secondly, longer follow-up durations would be beneficial to mitigate the impact of lead time bias. Lastly, further research exploring the correlation between tumor morphology and genetic risk factors is necessary.

In conclusion, echography is an applicable method to evaluate tumor configuration as well as tumor size, relations with ciliary body, optic nerve and sclera. The shape classification, as an independent factor, may provide better prognostic accuracy when combined with AJCC stage or LBD. We recommend integrating optimized tumor shape categories into the current prognostic models.

## Data Availability

The original contributions presented in the study are included in the article/supplementary material, further inquiries can be directed to the corresponding author/s.
